# Affective Computing and the Impact of Gender and Age

**DOI:** 10.1371/journal.pone.0150584

**Published:** 2016-03-03

**Authors:** Stefanie Rukavina, Sascha Gruss, Holger Hoffmann, Jun-Wen Tan, Steffen Walter, Harald C. Traue

**Affiliations:** 1 Department of Psychosomatic Medicine and Psychotherapy, Medical Psychology, Ulm University, Ulm, Germany; 2 College of Teacher Education, Lishui University, Lishui, P.R. China; University of Pécs Medical School, HUNGARY

## Abstract

Affective computing aims at the detection of users’ mental states, in particular, emotions and dispositions during human-computer interactions. Detection can be achieved by measuring multimodal signals, namely, speech, facial expressions and/or psychobiology. Over the past years, one major approach was to identify the best features for each signal using different classification methods. Although this is of high priority, other subject-specific variables should not be neglected. In our study, we analyzed the effect of gender, age, personality and gender roles on the extracted psychobiological features (derived from skin conductance level, facial electromyography and heart rate variability) as well as the influence on the classification results. In an experimental human-computer interaction, five different affective states with picture material from the International Affective Picture System and ULM pictures were induced. A total of 127 subjects participated in the study. Among all potentially influencing variables (gender has been reported to be influential), age was the only variable that correlated significantly with psychobiological responses. In summary, the conducted classification processes resulted in 20% classification accuracy differences according to age and gender, especially when comparing the neutral condition with four other affective states. We suggest taking age and gender specifically into account for future studies in affective computing, as these may lead to an improvement of emotion recognition accuracy.

## Introduction

Affective computing can be described as “computing that relates to, arises from or deliberately influences emotions” [1]. Therefore, it is essential to correctly identify and recognize these human emotional reactions in order to improve the interactions between digital devices and their users. People tend to manifest and communicate emotional reactions during human-computer interactions (HCI) that display similarities to emotions reported in human-human interactions (HHI) [2]. Similarities regarding these emotional reactions have been studied in detail [3]. There are only small discernible differences for, e.g., “disgust,” which is significantly more often reported during HHI, whereas “getting annoyed” is more frequently reported during HCI.

To improve HCI by adaptation to individual users’ needs and situations, a research project “SFB/TRR 62” (http://www.sfb-trr-62.de/) is currently pursuing the idea of a companion technology with personalized user models and automated recognition of mental states like emotions, dispositions and intentions. Such companion technologies should not be understood as technical devices, rather, as cognitive digital abilities to adapt individually to their users’ mental states, and trusted as supporting cognitive companion systems [4].

Overcoming this challenge of recognizing the emotional and dispositional states of a user in a robust manner and with high recognition accuracy, human-computer interactions would thus achieve a higher degree of quality. It would be possible to use such companion technologies as supportive digital companions, e.g., for people with special demands such as elderly individuals, or as elaborated in Walter et al (2013): “its application potential ranges from novel individual operation assistants for the technical equipment to a new generation of versatile organization assistants and digital services and, finally, to innovative support systems, e.g., for patients in rehabilitation or people with limited cognitive abilities” [5]. Companion technology goes beyond assistive technology if the recognition of users’ mental states is used to adapt to and support the users’ goals through meaningful feedback.

Due to the fact that affective computing is a very broad area of research, only a limited number of elements of the general goals have been considered in the past, e.g., measuring psychophysiological parameters in HCI, as well as the process of feature extraction and classification of emotions. However, the impact of different subject-specific variables such as gender, age, personality and gender role in the process of classification and feature selection have rarely been taken into account.

### 1. Emotions and Affective Computing

When conducting studies in affective computing it is important to measure all crucial behavioral and physiological changes during a specific emotion or emotional event. Yet it is also important to analyze different variables that have been reported to have an impact on the emotional reaction itself.

As Scherer (2000) and Gross and Feldman Barret (2011) demonstrate and sum up in two different articles, there are many theories pertaining to emotions and ideas in terms of how they evolve [6–7]. However, two main emotion theories can be differentiated that postulate either discrete emotions, e.g., basic emotions [8–9], or dimensional emotions allocated in a dimensional affect grid, e.g., according to the dimensions of valence, arousal and dominance [10–11]. In our point of view there are some benefits for the dimensional construct of emotions using physiological parameters in affective computing as they offer more varied emotional states than predefined conditions within the discrete emotions model.

Although many aspects of different emotion theories cannot be generalized into a sole definition of emotion and what Lindquist describes as a “hundred-year emotion war” [12], there are many aspects that have been accepted by several emotion researchers: emotions are complex phenomena consisting of a variety of cognitive, behavioral and physiological activations [4, 13–14]. The changes and activations occur almost simultaneously but with different time delays, thus the content should not be strictly evaluated in a chronological order. First of all, the subjective experience changes and a cognitive assessment of inner and/or outer stimuli can be detected. As emotions are motivational-related they also activate physiological parameters to enable the human in an approach-avoidance based manner. This behavior involves the human displaying reactions that contribute to approaching the stimulus if the latter appears positive and rewarding; conversely, the human avoids the stimulus if it appears negative and harmful. Both approaches include changes in expressive behavior (e.g., facial expressions, gestures or speech) and changes in physiology (reactions in central and autonomous nervous system and endocrine system).

The measurement of emotions can therefore be realized, in principle, by the measurement of these components. Each of these changes can be measured with different methods, e.g., measurement of peripheral physiological recordings (electromyography, skin conductance, heart rate, respiration, etc.), subjective ratings (e.g., questionnaires) or video analysis. Combining all parameters and modalities, a fusion-based multimodal classification should ideally be able to detect and recognize emotions; see 1.2.

### 2. Machine Learning and Emotion Classification

Most researchers in the field of affective computing perform emotion detection and recognition using so-called classification methods. These classifications and their underlying basics and rules are incorporated into the field of machine learning.

In general, machine learning systems can be captured as artificial systems that learn from known data and are able to find and recognize characteristic patterns. They deliver a model that can be used to classify unknown input data into a category (class) after a “training” phase. There are several different classification methods, e.g., neuronal networks or logic-based networks. However, the usage of neuronal networks and support vector machines (SVM) have been reported in literature as obtaining the highest classification results when using multidimensional data [15].

How a SVM works is explained in detail in the Gruss et al. article regarding the classification of pain [16]. To this end, we refer to the following passage: “The goal of an SVM is to develop a predictive model based on the given training samples (x_i_, y_i_), with x_i_ being a feature vector and y_i_ its associated class label. This model can subsequently be applied to an unlabeled test dataset to assign a particular class to each sample. With the aid of the feature vectors x_i_, the SVM […] searches for an optimal hyperplane with maximum margin in the feature space that separates the feature vectors of one class from feature vectors of the other. The hyperplane thus serves as the decision function [for unknown data]. If the linear separation is not possible in the original feature space, all training vectors can be transformed to a higher dimensional space until the SVM finds a dividing hyperplane” [16]. For more information the reader may refer to [17].

There are several different entities that can be used for classifying emotions, e.g., physiology, audio or video. It is assumed and already proven that a fusion of all these mentioned signals leads to higher accuracy rates as opposed to relying on one single channel [18]. Psychophysiological signals, however, do have the advantage of being continuously available even when other signals—for instance, video analyses—are not feasible due to poor lighting. Additionally, physiological parameters are more difficult to control, e.g., in terms of regulation mechanisms and can be measured as “honest signals” in interactions [19]. Therefore, in the current study we focused only on physiological signals to detect the influence of subject-specific variables (age, gender, personality, and gender roles), which have thus far been neglected in the field of affective computing. We believe this is an essential step to improve emotion detection and recognition in prospective studies.

### 3. Emotions and the Influence of Gender, Age, Personality, and Gender roles

Although some emotion-related literature presents results showing subjects variables as an influence of psychophysiological reactions during an emotional state, the research of affective computing has not considered and analyzed these influences in detail. Therefore, we shall attempt to provide a short overview of the influence of subject-specific variables in emotion research: gender, personality, age, and gender roles.

At first, our focus will be placed on the impact of gender on psychophysiological reactions and their potential influence on classification results. As mentioned in 1.1., emotions induce motivational-related behavior, eliciting approach-avoidance reactions. Yet there seems to be a gender difference: in their study, Bradley and colleagues found different motivational-related reactions in response to various valenced picture materials [20]. They concluded that males react more responsively in a physiological manner to positive stimuli with an appetitive motivation, whereas women react more to negative stimuli which activate a defensive motivation, leading to an avoidance behavior. These gender-specific differences were also found and reported for music stimuli [21] and for picture induction measured on the central nervous system, e.g., an EEG-measurement showed higher N200 amplitudes for females in response to negative pictures [22] and greater extrastriate activity for males during erotic stimulation, measured with an fMRI [23].

Considering gender differences in psychophysiology, researchers encounter the often neglected influence of sexual hormones on emotional reactivity and physiology. Only a few studies combine these two aspects (gender and sexual hormones) and report specific gender differences depending on the female menstrual cycle (corresponding with hormonal fluctuations) that also have been reported in a review to have an impact on physiological reactions to emotional stimuli [24]. For instance, Sakaki and Mather cite a higher physiological reactivity of follicular women in response to positive stimuli, which is purported to be mediated by a higher concentration of estrogen, whereas women in the luteal phase show higher physiological reactivity in response to negative stimuli mediated by a higher progesterone concentration. Both stimulus-dependent reactions are similar to the postulated appetitive-defensive motivational behavior.

Within the research field of affective computing, the quantity of literature concerning a potential influence of gender or further sexual hormones on classification accuracy is very limited. To our knowledge, only few studies have investigated the influence of gender on affective computing, thus little is known about this influence. Regarding the usage of solely audio information to recognize emotions, Vogt and André report an improvement of up to 3% when considering gender within the classification process [25]. Rukavina et al. showed an improvement of up to 8% of classification rates using only two physiological parameters when considering gender [26]. Although these mentioned outcomes seem to be low compared to the classification accuracy overall, they also indicate an improvement that offers the potential for further improvement through inclusion of other subjective, specific variables.

The second section outlines the impact of personality on physiology. Personality theories attempt to describe differences between individuals in “affect, cognition, and behavior, across situations and over time” [27]. According to Stemmler, personality operates in tandem with stable experiences and behaviors [28]. More precisely, certain combinations of emotional, attitudinal and behavioral response patterns of an individual define their personality. Different personality theorists present their own definitions, explanations, and specific personality traits: e.g., a biologically based and explained behavioral activation and inhibition system (BIS/BAS)[29] or the often employed five-factor dimension-model measured using the NEO/FFI [30]. What proves interesting is that several studies in the past showed how psychophysiological differences can be explained on the basis of various personality traits and their values. For example, Balconi and colleagues reported high BAS subjects to be more responsive to positive emotions and high BIS subjects to be more responsive to negative and arousing emotions measured with skin conductance reactions [31]. Additionally, they report positive correlations between the subjective ratings for positive ratings and high BAS subjects and negative ratings for high BIS subjects [31]. These results indicate not only that the subjective rating is influenced by our personality, but also by—or in accordance with—our physiological reactions and differences in response to different valenced stimuli. Similar results are reported in the study conducted by Koelsch and colleagues [32]. They report correlations between cardiac amplitudes and personality traits [32], indicating and supporting findings for the postulated connectivity between personality and its impact on psychophysiology.

To our knowledge, literature regarding personality and affective computing is likewise very limited. To date, it has focused more on classifying personality traits from, e.g., audio signals [33] or endeavored to inject personality into “lifelike characters” in order to improve HCI [34] as opposed to being considered as a variable influencing the emotion classification process.

In addition to gender and personality, age is also reported as a subject-specific variable that has an impact on physiological reactions during emotional states. Elderly people were reported to have lower physiological reactivity measured with electromyography and skin conductance [35]. Levenson et al. showed the same tendency of lower physiological reactions measured through heart rate changes and finger temperature [36]. Age can be enqueued to the neglected variables such as gender and personality in affective computing, although it is already recognized and discussed to play a role for the future population due to demographic changes. At this juncture we believe it is necessary to investigate the difference between young and elderly individuals to improve classification processes.

As a final possible important subject-specific variable (in this study), gender roles shall be taken into account. This construct is more of a reference to the socially-lived gender within a social role, compared to the biological difference. As discussed, gender role was found in only a few studies to be influential on emotional reactions, particularly with respect to facial expressiveness [37–38]. Literature regarding this influence is likewise rare. To date, this variable was so far only considered in the field of affective computing when the attitude towards computers was tested and analyzed. For example, Colley and colleagues reported a positive correlation of masculinity with positive computer attitudes [39].

To summarize the above-mentioned studies and outcomes, it can be concluded that although the listed subject-specific variables may have a positive outcome on classification results, most of them have been neglected within the research field of affective computing. However, we do believe that if there are differences between emotional reactions and those subject variables that they will also have an impact on the classification process.

## Aims and Hypotheses

The current study analyzed the impact of user-specific variables on the affect classification during emotion inductions in a simulated HCI. In detail, gender, age, personality traits, and gender roles are considered to play a role on psychophysiological reactions and are therefore analyzed with correlation analyses in Step 1. In total, 20 physiological features were extracted from two EMG channels (Musculus corrugator supercilii and Musculus zygomaticus major), skin conductance, and heart rate variability.

In Step 2 all significant variables were used to select specific subsamples for classification of different affects during the emotion elicitation task. Afterwards an analysis of the amount of the selected features should provide insight about the most important features and if there are determinable subsample specific feature-groups.

Our hypothesis in general suggests that subject-specific classifications improve classification accuracies. In detail, we hypothesize gender, personality dimensions, e.g., neuroticism, extraversion, BIS dimension and BAS dimension, age, and gender roles to be influential with regard to physiological reactions and, as a consequence, classification accuracy.

## Procedure

To induce core affects we used standardized affective picture material. Specifically, we utilized pictures from the International Affective Picture System [40–41] as well as Ulm pictures [42] to represent the whole VAD (valence, arousal, dominance) space according to their ratings. To intensify the elicitation we used a prolonged presentation [43–44]. This prolonged paradigm was chosen since affective reactions may occur for longer periods in ongoing interactions, e.g. human-computer interactions.

**Fig 1:**
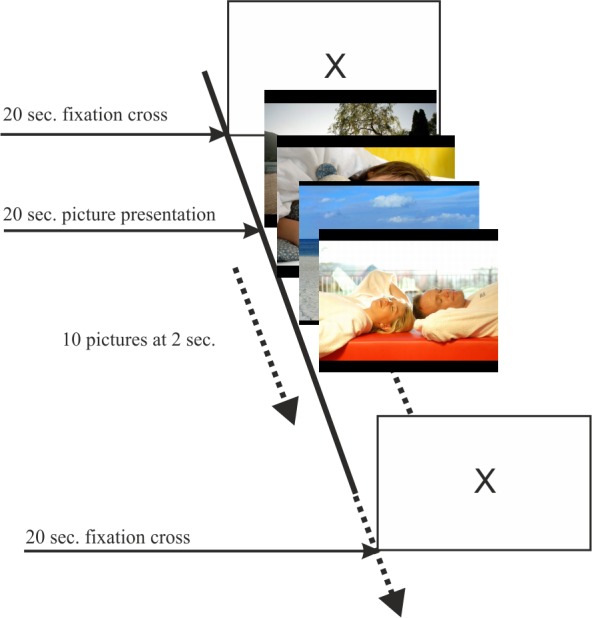
Emotion elicitation task. Every picture presentation was begun by presenting a fixation cross for 20 seconds. Afterwards, 10 pictures belonging to the same core affect were displayed without a pause for two seconds each.

All pictures with similar ratings in terms of valence and arousal were combined into one of the five core affects: neutral, HVHA (high valence and high arousal), HVLA (high valence and low arousal), LVHA (low valence and high arousal) and LVLA (low valence and low arousal). Each core affect was represented by a block of 20 pictures, wherein each picture was displayed for two seconds and without a pause. There were two blocks for each core affect; in total, 100 pictures were used for the induction. Between each block, a fixation cross was displayed for 20 seconds to return tentative physiological reactions to the initial baseline. For a graphical explanation see Fig 1.

### 1. Subjects

All subjects were right-handed recruited via an advertisement posted at the University of Ulm and at the University of Magdeburg. All participants were financially remunerated for participation. They were healthy and had normal vision or corrected normal vision.

The study was designed in accordance with the ethical guidelines set out in the WMA Declaration of Helsinki (ethical committee approval was granted: (#245/08-UBB/se). The study was approved according the ethics committee of the University of Ulm (Helmholtzstraße 20, 89081 Ulm, Germany). All participants provided a written informed consent to participate in this study.

Our sample consisted of a total of n = 127 subjects. Since we sometimes received partially filled out questionnaires (NEO-FFI, BSRI, BIS/BAS, as described in the following section) and due to signal artifact reduction analyses, our final sample consisted of 100 subjects (n = 64 women and n = 36 males) between 20 and 75 years old (average age 38.57 years, SD = 19.28) which were analyzed for their physiological reactions as well as gender, age, personality dimensions, and gender role.

### 2. Physiological Recording

**Fig 2:**
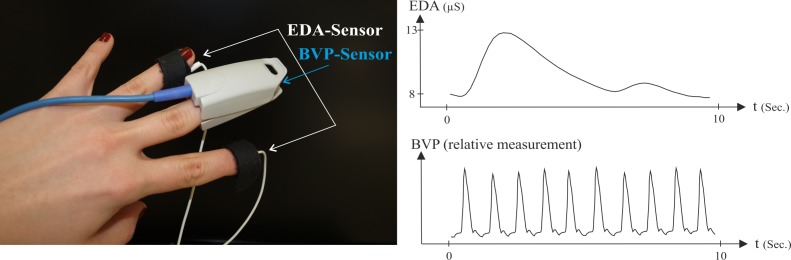
Placement of the sensors BVP and EDA: Both sensors were attached to the left hand (non-dominant hand). On the right side you can see a sample recording of both signals. The BVP signal is a relative measurement and is therefore processed without a specific unit.

All three physiological components—heart rate variability (HRV), skin conductance, and facial EMG—were recorded with a NeXus-32 (NeXus-32, Mind Media, the Netherlands) and the trigger data was recorded with the software Biobserve Spectator (BIOBSERVE GmbH, Germany). HRV information was measured with a BVP (blood volume pulse) sensor. Via infrared light the sensor measured the blood volume running through the blood vessels within each heart period, using a non-invasive technique (plethysmography). This sensor was attached to the left (non-dominant) middle finger of every right-handed subject; see Fig 2.

To measure the electrodermal activity (EDA), two electrodes were attached to the left index and ring fingers. By conducting a small, direct current through two Ag/AgCl electrodes, it is possible to record the conductivity changes induced by the sympathetic innervated perspiratory glands.

**Fig 3:**
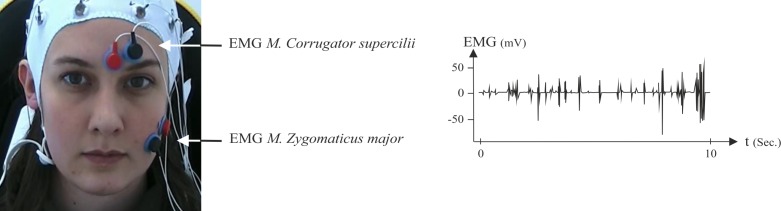
Placement of fEMG electrodes on the M. corrugator supercilii and M. zygomaticus major. On the right side you can see one example of a filtered EMG signal. (The individual in this picture has given written informed consent (as outlined in PLOS consent form) to publish this photo).

The facial EMG (fEMG) activity was recorded by using bipolar miniature silver/silver chloride (Ag/AgCl) skin electrodes of 4 mm diameter. Both electrodes were placed on participants’ left corrugator supercilii and zygomaticus major muscle regions (see Fig 3), according to the guidelines for fEMG placement recommended by Fridlund and Cacioppo [45]. Prior to each recording, the biosignals were visualized with the BioTrace software (appertained to the NeXus-32) and corrected, if necessary, to avoid bad signals or other unwanted influences.

## Questionnaires

All participants were required to fill out two questionnaires regarding personality and their gender role: the NEO-FFI, BIS/BAS and the BSRI.

The NEO-FFI [30] is an established questionnaire to measure the five different personality traits: agreeableness, openness, extraversion, neuroticism and conscientiousness. The NEO-FFI consists of 60 questions, 12 for each trait on a five-point Likert scale. This model is a data-based, cross-sectional and empirically proven model.

The BIS/BAS questionnaire was developed on the basis of Gray’s personality theory [46]. This theoretical framework was designed to explain differences in emotional behavior among individuals and consists of three systems: BIS (behavioral inhibition system), BAS (behavioral activation system), and the FFS (flight/fight system, which is not further considered in this study). The BIS/BAS questionnaire measures two parts of the motivational system [29]. On the one hand, the BAS scale measures the drive/impulse to go for the stimulus, the reward sensitivity, and the fun-seeking behavior. On the other hand, the BIS system mediates the sensitivity to signals of punishment and non-reward behavior. The questionnaire consists of 24 questions pertaining to the subjects’ feelings, activating the BIS or BAS system, which can be answered on a 4-point Likert scale.

The Bem Sex Role Inventory (BSRI) was developed in 1974 [47]. BSRI is a measure of masculinity-femininity and gender roles. The questionnaire consists of 60 personality characteristics that are either masculine, feminine, or filler items (20 items for each). Participants rate themselves based on a 7-point Likert scale.

## Signal Processing and Feature Extraction

All extracted features are baseline corrected and normalized individually. The features were calculated and extracted with reference to Kim and André as well as Picard’s study [48–49].

### 1. Heart Rate Variability

Prior to calculating the HRV it was necessary to determine the inter-beat interval of successive heart beats (NN-interval). For this step we developed a Matlab script that extracted these intervals almost automatically; however, every segment was displayed on the screen for visual correction and to provide the opportunity to correct the NN identification or to delete the file.

Extracted HRV features:

1 mean of the NN-Intervals
2 standard deviation of the NN-Intervals
3 mean of the heart rate
4 standard deviation of the heart rate
5 root mean square of successive differences (RMSSD [ms]):

$$\text{RMSSD}=\;\sqrt{\frac{1}{\text{n}-1}\sum_{\text{j}=1}^{\text{n}-1}({\text{NN}}_{\text{j}+1}-{{\text{NN}}_{\text{j}})}^{2}}$$

We did not use frequency domain features, due to problems with the validity of short HRV recordings [50].

### 2. Facial Electromyography

The raw facial EMG of the corrugator supercilii and zygomaticus major were filtered offline using a 20–250 Hz bandpass Butterworth filter (order = 4) to exclude motion- related components and an adaptive filter was applied to accommodate the 50 Hz power line interference. The signals were then rectified and smoothed by the root mean square (RMS) technique using a 125 ms sliding window. Facial EMG changes were derived from subtracting baseline activity (i.e. the mean of the RMS of two seconds before each picture block onset) from the respective picture block viewing periods (i.e. the mean of the RMS). Subsequently, we standardized (i.e. Z score) EMG changes within each participant and within each site according to [45]:

6 mean of the amplitudes of M. corrugator supercilii
7 mean of the amplitudes of M. zygomaticus major

### 3. Skin Conductance Level

The SCL signal was filtered offline with a 0.2 Hz low pass filter to smooth the signal. Additional preprocessing steps were unnecessary due to the favorable quality.

Extracted SCL features:

8 mean of the raw signal:
  $$\text{SCL}\_mean=\frac{1}{N}\sum_{n=1}^{N}{\;(X}_{n})$$
9 standard deviation of the raw signal:
  SCL_std=(1N−1∑n=1N(Xn−μx)2)12
10 mean of the first difference of the raw signal:
  $$\text{SCL}\_\text{fd}\;=\frac{1}{\text{N}-1}\sum_{\text{n}=1}^{\text{N}-1}\;|{\text{X}}_{\text{n}+1}-{\text{X}}_{\text{n}}|$$
11 mean of the first differences of the normalized signal:
  $$\text{SCL}\_\text{fd}=\frac{1}{\text{N}-1}\sum_{\text{n}=1}^{\text{N}-1}\left|{\tilde{\text{X}}}_{\text{n}+1}-{\tilde{\text{X}}}_{\text{n}}\right|$$
12 mean of the second difference of the raw signal:
  $$\text{SCL}\_\text{sd}\;=\frac{1}{\text{N}-2}\sum_{\text{n}=1}^{\text{N}-2}\;|{\text{X}}_{\text{n}+2}-{\text{X}}_{\text{n}}|$$
13 mean of the second difference of the normalized signal:
  $$\text{SCL}\_\text{z}\_\text{sd}=\frac{1}{\text{N}-2}\sum_{\text{n}=1}^{\text{N}-2}\;|{\tilde{\text{X}}}_{\text{n}+2}-{\tilde{\text{X}}}_{\text{n}}|$$
14 amount of amplitudes with increasing slope:
  Maxima=amplitude>0.05μS
15 amount of amplitudes with decreasing slope
  Minima=amplitude>−0.05μS
16 total amount of all Extrema
  $$\text{Extrema}={\text{n}}_{\text{Maxima}}+{\text{n}}_{\text{Minima}}\;\text{Extrema}:\;\text{n}\_\text{Maxima}+\text{n}\_\text{Minima}$$
17 mean amplitude of maxima
18 mean amplitude of minima
19 integral of the signal
20 slope of regression

## Feature Selection and Reduction

After the feature extraction, a feature reduction method was applied according to [16]. By deleting features that correlated 0.95 and -0.95 respectively, we attempted to eliminate features with redundant information, to prevent classifying noise and as Liang and Zhao describe it “Re-moving or reducing these irrelevant or redundant features is very important because they may deteriorate the performance of classifiers.” [51]. At the end of the reduction process the feature list used for classification consisted of 15 features (see aforementioned features) and the following features were excluded: (3), (4), (11), (12), (13).

All classification processes were extended by additional feature selection processes to compare the highest recognition rates. In general, feature selection methods are applied to optimize recognition accuracies by using a subset of features conveying the important information. Several different selection processes are utilized [52]. In the current study we limited the feature selection process to forward selection and backward elimination. We tested both feature selection methods to analyze the impact of subject-specific variables on classification accuracies (independent of the selection process) and report only the result of the classification and feature selection method that achieved the higher accuracy.

## Correlations of user attributes and psychophysiological features

The correlation analysis was conducted for all subject-specific variables, age, personality, and gender roles. Because gender differentiation has already been shown to be beneficial for a classification process [26], we did not include gender in the correlation matrix.

We conducted several correlation analyses between the psychophysiological extracted features and age, male role, female role, extraversion, neuroticism, BIS and BAS with a Bonferroni-corrected significance level of p < 0.007.

## Classification Procedure

For classification of different psychobiological affective states we chose a SVM, as they have previously been proven to be very effective [53–54] and to maintain enough flexibility with regard to their main parameter optimization [55]; see Section 1.3. For more details on SVM the reader may refer to [17].

All classification accuracies in the current paper were calculated by using a “batch validation.” This validation process enables subject leave-one-out results and is therefore of higher validity.

## Results

### 1. Correlation Analyses

Age was the only component found to correlate significantly with most SCL features during different affective states and one HRV feature, namely RMSSD, during condition LVLA; see Table 1. There were no significant correlations between psychobiological features and gender role or personality scores after Bonferroni correction.

Therefore, all following classification results were constructed by splitting the sample into gender and age—specific subsamples: young females n = 43 (mean age = 24.79 SD = 4.23); young males n = 22 (mean age = 23.86 SD = 3.66); elderly females = 21 (mean age = 60.62 SD = 5.17) and elderly males = 14 (mean age = 69,6 SD = 5.2); see 9.2.

**Table 1:** Correlation of user specific variable (age) and features*.

Feature and Condition	Age
SCL_mean_raw_neutral	r = -0.466; p = 0.000
SCL_mean_raw_HVHA	r = -0.394; p = 0.000
SCL_mean_raw_LVHA	r = -0.462; p = 0.000
SCL_mean_raw_HVLA	r = -0.460; p = 0.000
SCL_mean_raw_LVLA	r = -0.458; p = 0.000
SCL_std_raw_neutral	r = -0.250; p = 0.006
SCL_std_raw_HVHA	r = -0.319; p = 0.000
SCL_std_raw_HVLA	r = -0.246; p = 0.000
SCL_mean_fd_raw_neutral	r = -0.345; p = 0.000
SCL_mean_fd_raw_HVHA	r = -0.347; p = 0.000
SCL_mean_fd_raw_LVHA	r = -0.337; p = 0.000
SCL_mean_fd_raw_HVLA	r = -0.331; p = 0.000
SCL_mean_fd_raw_LVLA	r = -0.336; p = 0.000
SCL_slope_regression_HVLA	r = -0.346; p = 0.000
RMSSD_LVLA	r = 0,272; p = 0.003

*only significant results are reported;

r = Pearson´s correlation coefficient; p = significance level

### 2. Classification Results

As age and gender were found to have an impact on psychophysiological features, all classifications were conducted on a gender and age-specific basis. To elaborate, the subject groups were divided according to gender and their age group. Because we used two feature selection processes (backward elimination and forward selection) we reported the result with the highest accuracy as a rule.

An SVM was used for classifying three conditions according to valence—namely, neutral, positive and negative. Afterwards five affective states—namely, neutral, low valence and low arousal (LVLA), low valence and high arousal (LVHA), high valence and low arousal (HVLA) and high valence and high arousal (HVHA) were classified and compared. We also classified the classes of neutral vs. one of the four conditions (HVHA, HVLA, LVHA, LVLA). For the validation we used a batch validation. That is, all classification results are what are known as leave-one-subject-out results.

**Fig 4:**
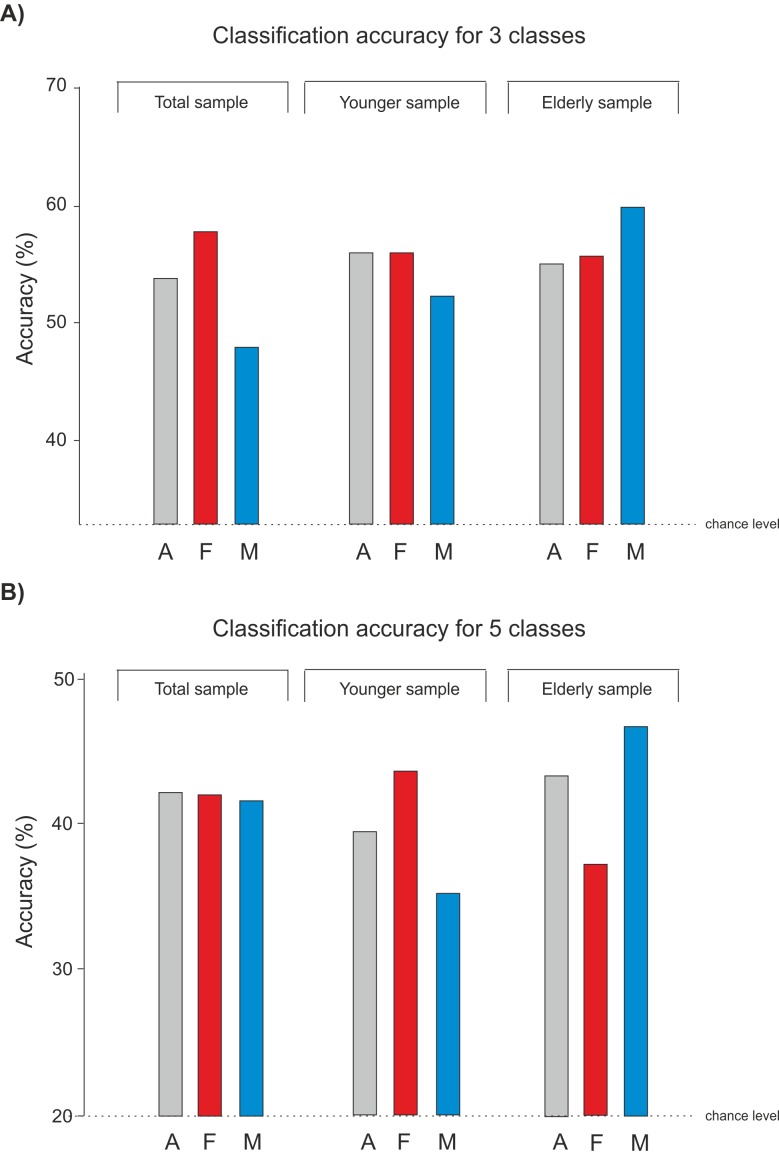
**Classification Results** for **A)** 3 classes (positive, neutral and negative) and **B)** 5 classes (HVHA, HVLA, LVLA, LVHA and neutral). All accuracies are presented for the different age groups (total sample, younger sample and elderly sample) and for A = All, F = Females and M = Males.

As is evident in Fig 4A), the chance level for three classes is at approximately 33.3%. The classification accuracy distinguishing between neutral, positive and negative was higher than by chance for every subject group. However, it is also apparent that the accuracy in the group of males is lower compared to all other accuracies.

The classification accuracies for distinguishing all five affective states in Fig 4B) show that young males achieved lower accuracies than the elderly male group. By contrast, young females show higher accuracies than the elderly female group.

**Fig 5:**
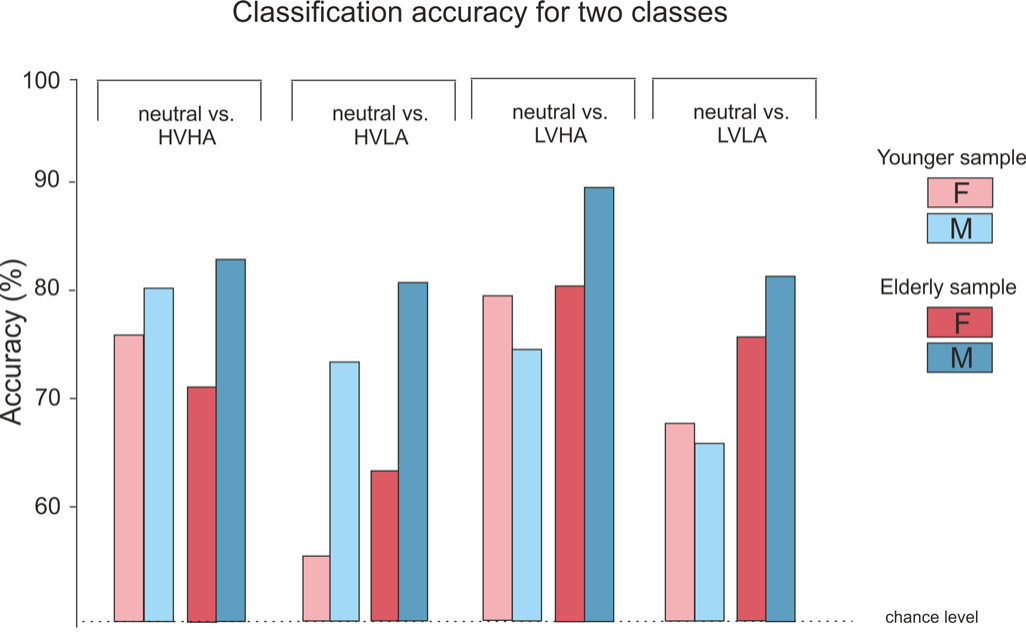
Classification results for 2 classes: HVHA vs. neutral, HVLA vs. neutral, LVHA vs. neutral and LVLA vs. neutral.

For the comparison of all affective states compared to the neutral state, Fig 5 shows the accuracy differences for each class comparison. In general, the group of the elderly males achieves the highest classification results. Young females achieve the lowest classification accuracy for distinguishing between neutral and HVLA. It is also clear that women in both age groups achieve higher accuracies distinguishing between neutral and LVHA, whereas males achieve higher accuracies in distinguishing between neutral and HVHA.

Collectively, the classification results from subject groups according to age and gender show accuracy differences of up to 20%.

An analysis of the selected features for all conducted classifications reveals the following results, see Table 2: Specifically the feature mean amplitude of M. corrugator supercilii was selected for 26 times and made the first place.

**Table 2:** Ranking of selected features for all subject specific classifications.

Feature	Signal	Amount	Ranking
mean_amp_Corr	fEMG	26	1
SCL_slope	SCL	24	2
SCL_std	SCL	23	3
mean_amp_Zyg	fEMG	23	4
mean_RR	HRV	20	5
SCL_mean_amp_max	SCL	19	6
SCL_mean_amp_min	SCL	19	7
SCL_fd	SCL	18	8
SCL_mean	SCL	17	9
SCL_n_maxi	SCL	14	10
SCL_integral	SCL	14	11
std_RR	HRV	11	12
SCL_n_ min	SCL	11	13
SCL_n_extrema	SCL	11	14
RMSSD	HRV	8	15

**Fig 6:**
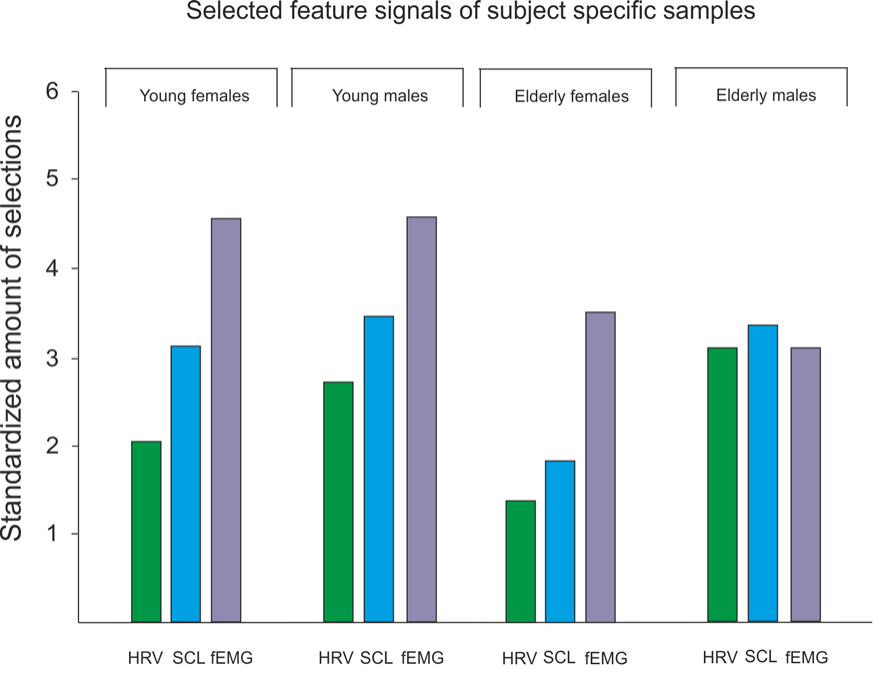
Comparison of selected features (belonging to one signal group) for each subject specific subsample.

To look for sample-specific feature groups we standardized the amount of the selected features, pertaining to one signal group, and compared them, see Fig 6. selection of each signal.

As Fig 6 shows, the signal information of fEMG is often selected especially for young females, young males and elederly males, followed by the information of SCL and HRV. Interestingly, the sample of elederly males do have an almost equally

## Discussion

Since Rosalind Picard published her first article on affective computing and its aims and visions [1], much effort has been placed on finding special features with which computers and digital devices are enabled to identify users’ emotions and dispositions in human-computer interactions, by means of machine learning processes. The ability to build empathetic digital devices would enhance empathy and, according to Janssen’s description, “this could improve our health and well-being and greatly improve our future societies” [56].

To address and overcome this obstacle, there are many different possibilities of information that can be considered for identifying emotions as they are multidimensional phenomena (see 1.1), e.g., facial expressions, speech or physiology. The advantage of physiologically measured signals is not only their continuous availability but also their potential for transmitting “honest signals” during an interaction as asserted by Alex Pentland [19].

Although within emotion research several subject-specific attributes were reported to be influential on emotional reactivity (see 1.3), variables including age, gender, personality, and gender roles have not been considered as such in affective computing. In this current study, a standardized emotion induction procedure with presentation of pictures from the IAPS [57] was used to analyze the effect of user- specific variables on the classification process. For this purpose, we analyzed the impact of age and personality dimensions measured with the BIS/BAS questionnaire, the two dimensions of neuroticism and extraversion from the NEO-FFI questionnaire as well as the gender roles (femininity and masculinity). The subject-specific variable gender was not included in the correlation analysis but in the classification process instead, as it has been reported to be beneficial for the classification performance [25–26]. All variables (excluding gender) were correlated with all extracted features during each of the five affective states: neutral, HVHA, HVLA, LVLA and LVHA. A significant influence was found only for age, especially for the skin conductance features.

This result was quite surprising especially since an effect of personality has been reported in several studies in the past (see 1.3). One explanation, in our point of view, could involve the high significance level due to the Bonferroni correction. Another explanation could be the different designs from the studies reported in 1.3.: most of them used a short picture representation time of 6 seconds. Contrastingly, we used a prolonged presentation of different pictures with similar ratings, which indicates that possible temporal changes within the prolonged stimulation are neglected and not represented by the extracted features. In addition to the disparate experimental design, the analysis of the impact of personality on psychophysiological reactions differed in a way that, e.g., Koelsch and fellows report a statistically significantly different cardiac parameter between people related to neuroticism and positive emotions [32]. However, they conducted these analyses by splitting the subject sample into quartiles and compared only the lowest and highest quartiles (extreme group comparisons). Extreme group comparisons are sometimes ambiguously discussed in literature, e.g., MacCallum et al. critical article regarding dichotomization, as they lead to a loss of information which can result in an overestimated difference between subjects [58]. In our study, we therefore conducted correlation analyses comprising all subjects, since we aimed to consider only meaningful variables for our classification analysis and prevent unnecessary subject splits resulting in small subject samples.

Although we did not record ratings concerning our induction material (e.g. with a SAM rating) [59], the induction appeared to be strong enough to elicit physiological reactions enabling the SVM to classify the different affective states distinctly above chance level; see Figs 4 and 5. This effect is substantiated by virtue of the batch validation in our classification.

Comparing the classification accuracies between the subject-specific samples, it can be observed that, in general, the separation of the subject samples according to gender and age leads to an improvement of classification results depending on the condition. One interesting outcome is that the elderly male group achieved the highest accuracy rates for all of the conditions. Our explanation for this outcome is that older males seem to react more consistently, and in such a way that the SVM could differentiate optimally between the different affective states. However, an additional reason could be attributed to the fact that this subgroup consisted of only 14 subjects.

Another interesting finding constituted the gender context specific accuracy differences. Females tended to be better classifiable when we compared neutral vs. LVHA, whereas males better classify for neutral vs. HVHA. This finding is reminiscent of an often reported gender specific phenomenon, wherein females display more psychophysiological reactions to LVHA stimuli, according to the defensive motivation, and males to HVHA, according to the appetitive motivation [20]; see 1.3.

Concluding on the selected features for each subject specific sample we can say that in general fEMG seems to be most important feature followed by the SCL features and HRV features. Furthermore, for elderly males this selection seems to be more equally distributed, which substantiates the explanation of higher accuracy rates due to consistent physiological reactions. How important the fEMG information can be was also reported for the classification of pain [16]. Future studies should integrate this information and broaden the amount of extracted fEMG features.

On the basis of our findings, we conclude that when considering age and gender in affective computing, it is not only necessary to include the individual aspects of users, but also that these aspects are meaningful since classifiers achieve higher performance.

## Limitations and Outlook

Due to a low number of feature vectors for each subject (because of a low amount of stimuli), it was not possible to train and test the classifier on a subject individual level. A higher amount of trials would have provided the opportunity to investigate further individual classification differences (e.g. statistical analyses).

Considering the classification results, we were able to achieve high classification accuracies by using specific subject groups. But we believe that these accuracy rates can be still improved by adding additional features to the classifier, e.g. additional features extracted from each signal and also using additional signals like electroencephalograpy (EEG). Some studies show an advantage of the usage of EEG signals for classifying emotions in real-time as well [60], therefore we encourage futures studies in affective computing to broaden their signal range.

Concerning the feature reduction process, we decided to eliminate features manually that highly correlate with each other before we used them for classification. We are aware that there are more options and possible reduction methods e.g. a principal component analysis. Using a correlation analysis instead was just a part of our integrated automatic preprocessing workflow and in the end a matter of design.

Another problem concerned the interdependence of the subject groups. As a consequence, every specific subject group had a smaller sample size. Future studies should recruit subjects by specific criterion (age, gender) and one group comprising a variety of subjects in order to seek classification differences as a means of addressing the problem of changing classification sample sizes.

Although our subject sample was large, comprising 100 subjects, we would like to encourage future studies to analyze the effect of subject-specific variables in addition to the reported studies and variables for this research.

## Supporting Information

S1 File This excel file consists of 3 register tabs including Table A, the correlation analysis between the subject specific variables and the psychophysiological features. Table B, correlation analysis between the features for feature reduction. Table C, data for classification.
(XLSX)
